# The Laugh of a Child

**Published:** 1903-10

**Authors:** M. A. Weems

**Affiliations:** Columbus, Texas


### Correspondence.

### The Laugh of a Child.

Dr. Weems sends us the following, which we publish with pleasure:

COLUMBUS, TEXAS, September 25, 1903.

*Texas Medical Journal, Austin, Texas.*

DEAR DR. DANIEL: In glancing over the last pages of the "Red-Back," for July, 1903, my eyes fell upon "Laughs and Laughter," page 30. Some of the sentences are quite witty and true. The last part of the article, dealing with the physiological effects of laughter, being particularly good. I was forcibly reminded of Col. R. G. Ingersoll's "The Laugh of a Child," the wording of which is so beautiful, and the sentiment expressed so lofty and pure, that I quote it for the benefit of those who may not have seen it.

"The laugh of a child will make the holiest day more sacred still. Strike with hand of fire, O weird musician! the harp strung with Apollo's golden hair; fill the vast cathedral aisles with symphonies sweet and dim, deft toucher of the organ keys; blow, bugler, blow, until the silver notes do touch and kiss the moonlit waves, and charm the lovers wandering midst the vine-clad hills: but know, your sweetest strains are discords all, compared with childhood's happy laugh—the laugh that fills the eyes with light and every heart with joy. O rippling river of laughter! thou art the blessed boundary line between the beasts and men; and every wayward wave of thine doth drown some fretful fiend of care.

"O laughter! rose-lipped daughter of joy, make dimples enough in thy cheeks to catch and hold and glorify all the tears of grief."

Yours very truly,

M. A. Weems, M. D.

